# Cardioprotective Effects of Omega-3 Polyunsaturated Fatty Acids: Dichotomy between Experimental and Clinical Studies

**DOI:** 10.3390/md16070234

**Published:** 2018-07-10

**Authors:** Melissa Desnoyers, Kim Gilbert, Guy Rousseau

**Affiliations:** 1 Centre de Biomédecine, Hôpital du Sacré-Cœur de Montréal, Montréal, QC H4J 1C5, Canada; melissa.desnoyers.1@umontreal.ca (M.D.); kim.gilbert3@gmail.com (K.G.); 2 Département de Pharmacologie et Physiologie, Université de Montréal, Montréal, QC H3C 3J7, Canada

**Keywords:** omega-3, omega-6, myocardial infarction, cardioprotection

## Abstract

The high-fat diet of North Americans has a major impact on cardiovascular disease occurrence. Notably, fatty acids have been identified as important factors that could modulate such diseases, especially myocardial infarction (MI). Experimentally, omega-3 polyunsaturated fatty acids (PUFA) have demonstrated positive effects on cardiovascular disorders and have also shown cardioprotection by decreasing MI size. Although many animal experiments have clearly established the benefits of omega-3 PUFA, clinical studies have not reached similar conclusions. In fact, the findings of recent clinical investigations indicate that omega-3 PUFA play only a minor role in cardiovascular health. This dichotomy between experimental and clinical studies may be due to different parameters that are not taken into account in animal experiments. We have recently observed that the high consumption of omega-6 PUFA results in significant attenuation of the beneficial effect of omega-3 PUFA on MI. We believe that part of the dichotomy between experimental and clinical research may be related to the quantity of omega-6 PUFA ingested. This review of the data indicates the importance of considering omega-6 PUFA consumption in omega-3 PUFA studies.

## 1. Introduction

According to general belief, the consumption of omega-3 polyunsaturated fatty acids (PUFA) is good for health. Among the different known omega-3 PUFA, three long-chain omega-3 PUFA (20 atoms of carbon or more), including eicosapentaenoic acid (EPA: 20:5n-3), docosapentaenoic acid (DPA: 22:5n-3), and docosahexaenoic acid (DHA: 22:6n-3), exert biological activities [1].

One of the first pieces of evidence claiming that omega-3 PUFA have cardioprotective properties came from a study of Alaskan Eskimos who consistently present high serum cholesterol, but with virtually the total absence of cardiovascular diseases, indicating that the role of cholesterol may be altered by diet or genetic factors [2]. Examination of the diet of this population suggests that the consumption of omega-3 PUFA, found in fish and fish products, is elevated in comparison to other populations with higher rates of coronary heart disease (CHD) [3].

In recent years, however, different clinical trials and meta-analyses have concluded that omega-3 PUFA do not afford significant benefit to cardiovascular health [4,5,6,7]. The dichotomy between experimental and clinical research led us to hypothesize that the identification of different factors should be taken into account to reconcile both types of study.

Some investigations, performed in our laboratory, indicate that the presence of omega-6 PUFA could influence the outcome of omega-3 PUFA. In the current article, we will assess the data that brought us to this hypothesis.

## 2. Experimental Data on Omega-3 PUFA in Myocardial Infarction (MI)

In terms of MI, animal experiments have determined that dietary fish oil supplements could be beneficial to the ischemic myocardium and decrease infarct size. For example, rats that are fed with a diet containing 12% fish oil (18% EPA, 12% DHA) presented significantly smaller infarct size than their controls [8]. Another study indicated that dietary fish oil supplements (12% fish oil in total weight; 19% EPA and 13% DHA) exert cardioprotective effects against ischemia and reperfusion in isolated rat hearts [9]. Experiments on rabbits demonstrated that MI size was significantly smaller in an EPA-fed group (600 mg/kg/day for 2 weeks) than in the controls [10]. Similarly, DHA infusion (45 mg) in the pericardial space resulted in significant MI reduction in a pig model [11]. We have observed that the addition of DHA or EPA (5 g/kg) in a normal chow resulted in a smaller infarct size in a rat model of myocardial infarction [12]. This regimen corresponds to around 50 mg/kg for a human, using the factor of correction (6.2) suggested by the Food and Drug Administration. We also reported a study indicating that an omega-3 PUFA-enriched diet diminishes infarct size compared to an omega-6 PUFA-enriched diet [13], but to observe this protective effect, the ratio of omega-3/6 PUFA should be higher than 1:5, which is similar to the suggestion presented by Simopoulos [14]. Overall, it appears that a large consensus can be obtained in animal experiments favoring infarct size reduction with omega-3 PUFA.

**Figure 1:**
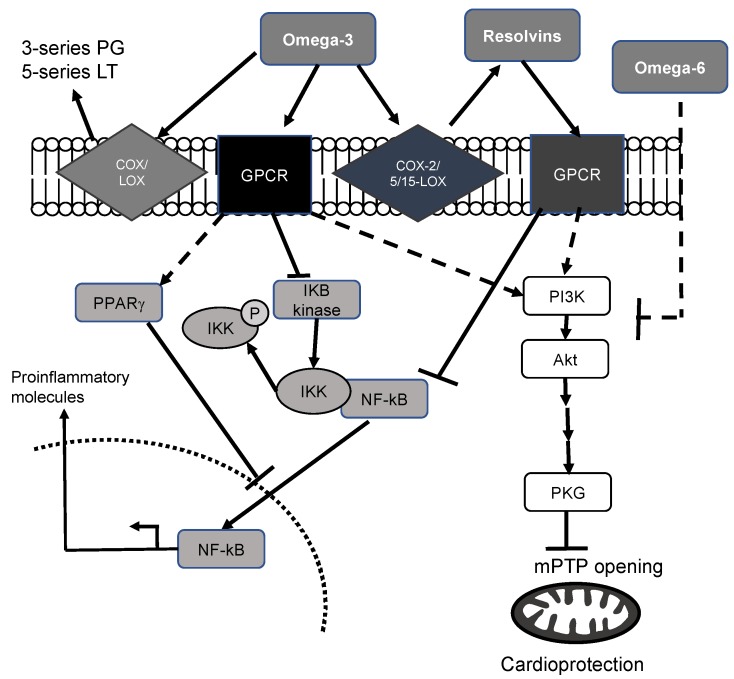
Possible mechanisms that explain the cardioprotective effects of the omega-3 PUFA and their metabolites. Dashed lines represent potential mechanisms. PG: prostaglandins; LT: leukotrienes; COX: cyclooxygenase, LOX: lipoxygenase; GPCR: G-protein-coupled receptors.

Although the mechanisms by which omega-3 PUFA are cardioprotective are essentially unknown, multiple hypotheses have been proposed in the literature (Figure 1). First, omega-3 PUFA have anti-inflammatory properties owing to their incorporation in cell membrane phospholipids [15], can be transformed into 3-series prostaglandins and 5-series leukotrienes. They have been identified as less aggressive biological molecules than Arachidonic acid-derived metabolites. Substitution of omega-6 PUFA in membranes by omega-3 PUFA results in major effects on inflammation [16,17,18,19]. It has been suggested that diminution of membrane omega-6 PUFA reduces “inflammatory metabolites”. The presence of EPA and DHA decreases cyclooxygenase-2 (COX-2) gene expression [20], although this has not been universally observed [21], by a mechanism involving the inhibition of NF-κB [20]. This effect results in a reduction of the inflammatory response, which could be beneficial for the ischemic myocardium [12,13,15].

Omega-3 PUFA may also induce cardioprotective signaling through the activation of G-protein-coupled receptors recently identified (GPR43, GPR120) [22,23]. According to the available data, it appears that DHA interacts with GPR120, resulting in interference with the nuclear factor-kappa B (NF-κB) pathway [22]. For instance, in macrophages activated by endotoxin, DHA inhibits IKB kinase as well as IKB phosphorylation and degradation, with the production of proinflammatory cytokines such as tumor necrosis factor-alpha (TNFα), an effect that is abolished in GPR120-knockdown cells. Interestingly, GPR120 gene transcripts have been upregulated in cardiac tissues with a high-fat diet (16% EPA, 9% DHA; [24]), indicating the presence of this receptor in cardiac tissues. Other possibilities have been pointed out to explain how DHA and EPA could inhibit NF-κB by interacting with peroxisome proliferator-activated receptor or by interfering with an early event before NF-κB activation [25].

We also have data indicating that a high omega-3 PUFA diet (ratio of omega-3/6 PUFA = 1:1) may be cardioprotective with the activation of the Akt pathway [13]. Studies performed to identify cardioprotective actions uncovered a biochemical pathway: reperfusion injury salvage kinase (RISK) [26]. The RISK pathway was originally referred to as the protein kinases Akt and Erk1/2. These kinases, when specifically activated at the time of reperfusion, confer powerful cardioprotection against lethal reperfusion injury culminating as a result of the opening of the mitochondrial permeability transition pore (mPTP), a key component in cardioprotection. Normally, the inner membrane of the mitochondria is virtually impermeable to metabolites and ions. In these conditions, the mPTP is closed [27]. Significant damage evoked by myocardial ischemia may induce mPTP opening and cytochrome C release, which could participate in cell death. Interestingly, it has been suggested that DHA can delay mPTP opening [28], contributing to the cardioprotective effect of omega-3 PUFA.

Another potentially beneficial outcome related to DHA could be attributed to their metabolites. When studying the resolution phase of inflammation, researchers identified resolvins (Rv) derived from EPA to form E series Rv (RvE1) and from DHA to form D series Rv (RvD1). RvE1 can be produced by EPA conversion to 18R-hydroperoxy eicosapentaenoic acid by aspirin-treated COX-2, followed by 5-lipoxygenase (5-LOX) [29]. Two receptors have been found to interact with RvE1: Chem-R23 and BLT1. RvE1 attenuates TNF-stimulated NF-κB activation through Chem-R23, whereas via BLT1, it behaves like an antagonist, assuaging leukotriene B4-dependent proinflammatory signals [30].

DHA is converted in vivo to RvD1 via a 15-LOX-initiated mechanism [31]. Aspirin-acetylated COX-2 generates 17R-hydroxy docosahexaenoic acid, which after sequential oxygenation by 5-LOX results in the production of 17-epi-RvD1, also known as aspirin-triggered RvD1. It has been suspected that COX-2 could also transform DHA into RvD1 without the presence of aspirin. Up to now, two GPRs have been shown to interact with RvD1: ALX-4 and GPR32. Similarly to RvE1, RvD1 attenuates TNF-stimulated NF-κB activation [32].

It has been demonstrated that RvE1 or RvD1 injection, in a rat [33,34] or pig [35] model of myocardial ischemia, significantly reduces infarct size, indicating the beneficial action of these metabolites on the myocardium via a mechanism that probably involves Akt.

Taken together, these data suggest that omega-3 PUFA and their metabolites act on multiple signalling pathways that could protect the myocardium from the ischemic insult.

## 3. Clinical Studies of Omega-3 PUFA

It is difficult to determine from clinical studies whether omega-3 PUFA have positive effects on cardiac health or not.

In a multicenter, double-blind, placebo-controlled trial, Kromhout et al. observed that a margarine supplemented with a combination of EPA and DHA (with a targeted additional daily intake of 400 mg of EPA–DHA) did not reduce the rate of cardiovascular events when patients who had an MI were receiving state-of-the-art (hypertension, thrombotic, and lipid-modifying) treatment [36].

Similarly, long-chain omega-3 PUFA supplementation (460 mg of EPA and 380 mg of DHA) had no effect on cardiovascular events in more than 3800 patients treated adequately for their conditions [37].

Bosch et al. reported that daily supplementation with 1 g of omega-3 PUFA (460 mg of EPA and 380 mg of DHA) had no significant influence on the rate of cardiovascular events in high-risk patients with more than 50% undergoing treatment with statins [38].

A meta-analysis that included 20 studies and 68,680 patients concluded that more than 1 g of long-chain omega-3 fatty acids supplementation was not associated with a reduced risk of all-cause mortality, cardiac death, and MI [7].

In contrast, according to Marchioli et al. [39], patients surviving a recent MI benefitted from low-dose omega-3 PUFA (around 860 mg EPA/DHA). Similarly, in hypercholesterolemic patients, daily use of EPA (1800 mg), in addition to a statin, decreased major coronary events by 19% compared to a control group given only statins [40]. At this point, it is important to note that statins alter the content of the serum omega-3/6 PUFA in hypercholesterolemic patients, which could contribute to the beneficial effect [41].

Reviewing 25 trials that evaluated coronary artery disease events, Harris et al. [42] noted that long-chain omega-3 PUFA, especially DHA, were significantly reduced in patients with CHD, supporting their beneficial role.

Overall, the favorable involvement of omega-3 PUFA supplementation is debated, as recently pointed out by a meta-analysis [43]. We must also consider that an increase in consumption of long-chain omega-3 fatty acids through the intake of fish or supplementation could result in different outcomes [44]. First, increasing the consumption of fish would probably result in a diminution of consumption of meat, which is not necessary in the case with the long-chain omega-3 PUFA supplementation. Second, there are some lipid microconstituents present in fish and fish products, apart from omega-3 PUFA, that exert cardioprotective properties, such as anti-Platelet-activating factor and antithrombotic activity [45,46], that could contribute to reducing cardiovascular risk.

## 4. Role of Omega-6 PUFA

**Figure 2:**
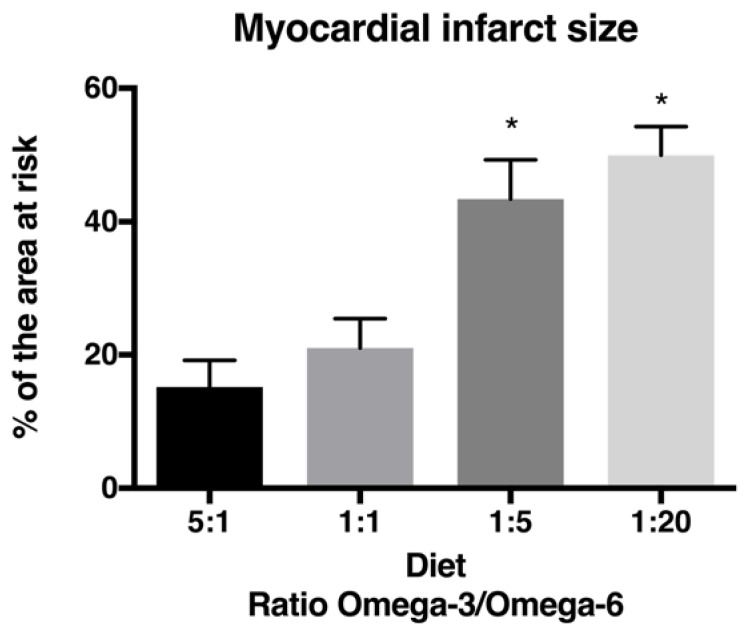
Infarct size expressed as percent of the area at risk (AR) in the presence of different diets with the different omega-3/6 ratios. * *p* < 0.05 (ANOVA followed by Bonferroni post-hoc test). (From [13,15]).

It is largely assumed that cardiac patients have high levels of omega-6 PUFA [47,48]. Meanwhile, their role in cardiovascular health remains controversial. According to different studies, omega-6 PUFA intake should represent around 5–10% of energy [49,50]. This value was derived from different studies indicating that such omega-6 PUFA intake was linked with CHD risk reduction [49]. A meta-analysis suggested that linoleic acid (LA) content was inversely related to CHD risk [51], and a clinical study concluded that high circulating LA levels were inversely associated with total and CHD mortality in older adults [52]. In contrast, it has been reported that AA and its metabolites possess proinflammatory and proaggregatory properties [53,54,55]. Others argue that omega-6 and omega-3 PUFA are metabolized by the same desaturation/elongation pathway and could thus influence long-chain PUFA content, which could reduce the beneficial effect of omega-3 PUFA. In a rat MI model, we observed that increased omega-6 and decreased omega-3 PUFA resulted in infarct size augmentation, indicating that the omega-3/6 fatty acid ratio may alter infarct size [15]. These findings are similar to what we have observed previously [13] and suggest that omega-6 PUFA may nullify the beneficial effect of omega-3 PUFA. The infarct size observed with the 1:1 omega-3/6 PUFA ratio is smaller than the one observed with the 1:5 ratio. However, a ratio higher than 1:1 did not afford greater protection, whereas ratios lower than 1:5 did not result in a greater infarct size (Figure 2).

**Figure 3:**
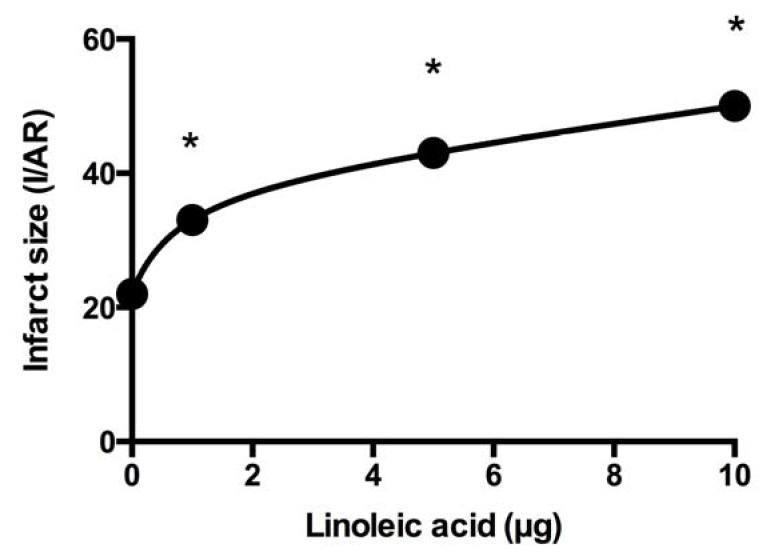
Infarct size (I) expressed as a percentage of the area at risk (AR) is increased with augmented LA dosage despite the presence of RvD1. * indicates difference with 0 µg LA. (From [56]).

At the same time, we studied the influence of omega-6 PUFA on the beneficial effect of RvD1 on infarct size. Our findings indicate that coadministration of LA (omega-6 PUFA) and RvD1 (0.1 µg) 5 min before ischemia results in a loss of protection compared to RvD1 alone. Infarct size is augmented with high LA doses (Figure 3). Since caspase-9 activity increases in the ischemic region with LA dose elevation, we hypothesize that it aggravates ischemia [56], and thus the administration of LA at the onset of reperfusion should not have an effect on infarct size.

**Figure 4:**
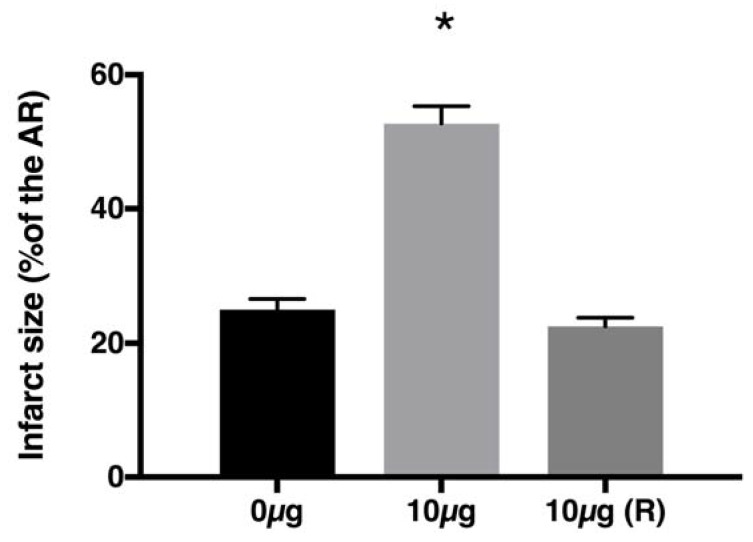
Infarct size in our MI model. RvD1 was injected 5 min before ischemia in the presence of 0 or 10 µg LA. We observed significantly increased infarct size with 10 µg vs 0 µg LA (* *p* < 0.05). However, when LA (10 µg) was injected at the onset of reperfusion (R), infarct size was similar to that obtained with 0 µg, suggesting that LA has an adverse effect during ischemia. (From [56]).

To evaluate this possibility, we injected LA at the onset of reperfusion, whereas RvD1 was delivered before the ischemic period. The results we obtained confirmed that LA affects ischemia intensity (Figure 4). Although not clinically relevant, it suggests that omega-6 PUFA levels present in the myocardium during ischemia may influence its intensity and thus may accelerate the rate of cardiomyocyte death.

It has been reported that LA produces a decrease in coronary flow, which may explain the harmful effect of LA in our context [53]. We have consciously used LA instead of arachidonic acid (AA) since it is well-known that AA induces a reduction of blood flow as well as other harmful effects for the cardiac tissue [57,58]. Moreover, we have previously observed that part of the positive effect of omega-3 PUFA on infarct size is related to the production of Rv [59]; inhibition of the transformation of omega-3 PUFA in Rv results in a larger infarct size.

By contrast, some metabolites of omega-6 fatty acids, such as prostacyclin [60] and lipoxin A [61], present anti-inflammatory and antithrombotic properties, which could be beneficial for the myocardium. However, in the context of myocardial infarction, we ignore whether the beneficial effects of these metabolites could overpass the harmful effect of other omega-6 fatty acid metabolites. Meanwhile, according to our results, we believe that in the context of myocardial infarction, a higher level of omega-6 fatty acids will not result in cardioprotection.

Overall, it is still difficult to draw conclusions about the “antagonist” effect of omega-6 PUFA against omega-3 PUFA in a context of myocardial infarction, since few studies have been published on the subject. However, populations that are large consumers of fish, such as the Inuit and Japanese, have a low mortality rate from ischemic heart disease [62,63,64]. This is also apparent in populations consuming a Mediterranean-type diet [65]. In these populations, large fish intake is usually associated with lower consumption of fat, especially animal fat and oils, which are rich in omega-6. However, the administration of omega-3 PUFA supplements without considering omega-6 PUFA consumed in the diet may be useless, according to our experimental data. Indeed, increasing omega-3 PUFA without any significant change in omega-6 PUFA consumption could result in failure.

## 5. Conclusions

The clinical and experimental results suggest that high concentrations of omega-6 PUFA could attenuate the beneficial role of omega-3 fatty acids in cardiovascular diseases. Due to the high level of omega-6 PUFA that we consume in industrialized countries, the addition of a supplement of omega-3 PUFA should not be enough to afford protection, and we must target a ratio higher than 1:5 of omega-3/6 PUFA. Future studies must also consider the consumption of omega-6 PUFA mainly when infarct size is evaluated.
